# Functional outcome after surgical treatment for spontaneous intracerebral hemorrhages: Development of the HeMAtOma score

**DOI:** 10.1016/j.bas.2025.104240

**Published:** 2025-03-21

**Authors:** Magnus Sættem, Ola Lønn Jenssen, Øystein Vesterli Tveiten, Stephanie Schipmann, Rupavathana Mahesparan

**Affiliations:** a Department of Neurosurgery, Haukeland University Hospital, Bergen, Norway; b Department of Biomedicine, University of Bergen, Bergen, Norway; c Faculty of Medicine, University of Bergen, Bergen, Norway; d Department of Clinical Medicine, University of Bergen, Bergen, Norway; e Department of Neurosurgery, University Hospital Münster, Münster, Germany

**Keywords:** Spontaneous intracerebral hemorrhage, Surgical treatment, Functional outcome, Risk factors, Predictive modeling

## Abstract

**Background:**

Spontaneous intracerebral hemorrhage (sICH) is a critical medical emergency associated with significant morbidity and mortality. The role of surgical intervention in improving functional outcomes remains a subject of debate.

**Objective:**

This study evaluates the functional outcomes of patients undergoing surgical treatment for sICH and identifies risk factors predictive of poor outcomes.

**Methods:**

A retrospective analysis of 100 patients treated surgically for sICH at Haukeland University Hospital between 2013 and 2022 was conducted. Baseline characteristics and clinical outcomes were collected. Functional outcomes were assessed using the modified Rankin Scale (mRS) at three months post-surgery. Independent risk factors for unfavorable outcomes (mRS ≥4) were identified through logistic regression.

**Results:**

The mean age was 65.5 years (56 % males). At three months, 54 % of patients had an unfavorable outcome, including a 23 % mortality rate. Independent risk factors for poor outcomes included age ≥60 years (OR 7.8, 95 % CI 1.684–36.3, p = 0.009), oral anticoagulant use (OR 10.4, 95 % CI 1.495–72.665, p = 0.018), and hemorrhage location in the basal ganglia (OR 18.5, 95 % CI 3.398–100.717, p < 0.001) or motor cortex (OR 8.6, 95 % CI 2.134–34.973, p = 0.003). These factors formed the basis of a new scoring system—the HeMAtOma score—which demonstrated good discriminatory ability (AUC 0.688) for predicting outcomes.

**Conclusion:**

Functional outcomes following surgical treatment for sICH remain poor in many cases. The HeMAtOma score provides a practical tool for predicting surgical outcomes, aiding clinical decision-making in emergency settings. Future prospective studies are needed to validate the score.

## Highlights

•**HeMAtOma score** developed for predicting functional outcomes after surgery for spontaneous intracerebral hemorrhage (sICH).
• **54 % unfavorable outcomes** (mRS ≥4) at three months post-surgery, with **23 % mortality.**
• **Key risk factors:** age ≥60 years, oral anticoagulant use, hematoma in basal ganglia, hematoma in motor cortex.
• **High-risk patients (score ≥ 50)** had an **84.2 % chance of poor outcome**, aiding surgical decision-making.
• **Retrospective study** requiring **prospective validation** for clinical implementation.

## Abbreviations

• sICH: Spontaneous intracerebral hemorrhage
• mRS: modified Rankin Scale
• CAA: cerebral amyloid angiopathy
• AHA/ASA: American Heart Association/American Stroke Association
• AVMs: arteriovenous malformations
• EVD: external ventricular drain
• OR: Odds ratios
• CIs: confidence intervals
• ROC: receiver operating characteristic
• AUC: area under the curve
• GCS: Glasgow Coma Scale

## 1 Introduction

Spontaneous intracerebral hemorrhages (sICH) are non-traumatic hemorrhages within the brain parenchyma, accounting for 10–15 % of all strokes (Sheth, 2022). These events present a critical medical emergency, with higher morbidity and mortality rates than ischemic strokes (Hemphill et al., 2001). Key risk factors include advanced age, male gender, cerebral amyloid angiopathy (CAA), and hypertension, with the latter being a major contributor to deep intracerebral hemorrhages (Jackson and Sudlow, 2006; An et al., 2017). For rapid diagnosis upon hospital admission, non-contrast CT is the preferred imaging method due to its wide availability and time efficiency (Kidwell and Wintermark, 2008). CT-angiography and contrast-enhanced CT can reveal active bleeding within the hemorrhage, known as the "spot sign," which is associated with hemorrhage expansion and poor prognosis (An et al., 2017; Hemphill et al., 2015; Wada et al., 2007; Cordonnier et al., 2018).

ICH treatment involves a comprehensive approach addressing both the primary injury from hemorrhage expansion and secondary processes leading to further neurological deterioration. Surgical hemorrhage removal offers potential benefits such as preventing mass effect and cerebral herniation, reducing intracranial pressure, and mitigating excitotoxicity and neurotoxicity from blood products (de Oliveira Manoel, 2020). However, the efficacy of surgery in ICH treatment remains controversial, as major randomized trials (STICH, STICH II, MISTIE III) have not definitively shown superiority over conservative management (Hanley et al., 2019; Mendelow et al., 2005, 2013).

American Heart Association/American Stroke Association (AHA/ASA) guidelines recommend surgical removal of cerebellar hemorrhage for patients with neurological deterioration, brainstem compression, or hydrocephalus from ventricular obstruction (Greenberg et al., 2022a). For supratentorial ICH, the usefulness of craniotomy for hemorrhage evacuation to improve functional outcome or mortality is uncertain. In case of clinical deterioration surgery might be considered as a lifesaving procedure (Greenberg et al., 2022a).

Spontaneous intracerebral hemorrhage (ICH) often entails long-term rehabilitation, with approximately half of all survivors requiring assistance with daily activities (Hemphill et al., 2015). Previous studies have reported 30-day mortality rates for sICH patients ranging from 16 % to 61 % (van Asch et al., 2010; Safatli et al., 2016; Sacco et al., 2009).

In emergency situations, accurately identifying patients who would benefit from surgery and allocating them to appropriate treatment remains challenging. This single-center study aims to assess the functional outcomes following surgery for ICH and identify potential risk factors associated with poor outcomes. The findings could potentially improve decision-making processes in ICH management.

## 2 Material and methods

### 2.1 Study population and patient data

We performed a retrospective observational study of patients who underwent surgical treatment for sICH at the Neurosurgical Department Haukeland University Hospital, Bergen, Norway, a tertiary neurosurgical referral center serving a catchment area of nearly one million individuals. We used our software for surgical planning and documentation (Orbit, Tietoevry Sweden AB, Sweden) to identify all patients who underwent surgery for sICH between January 2013 and December 2022 by searching for procedural codes and ICD-10 (International Classification of Diseases) codes.

Spontaneous ICH was defined as the manifestation of stroke symptoms in conjunction with evidence of parenchymal hemorrhage on cerebral CT without diagnosis of any underlying etiology. Exclusion criteria encompassed patients presenting with ICH resulting from trauma or secondary to cerebral aneurysms, arteriovenous malformations (AVMs), cavernomas, or other predetermined causes. Only patients aged 18 years and above were subject to analysis. We included only patients with available information on the Modified Rankin scale (mRS) at three months after surgery (Broderick et al., 2017). The mRS measures post-stroke disability on a scale from 0 (no symptoms) to 6 (death), with higher scores reflecting greater levels of disability.

Baseline characteristics such as age, sex, presenting symptoms, imaging and surgical details, patient's comorbidities and medication were obtained from the electronic medical records.

ICH volume was measured in the imaging program SECTRA (Sectra AB, Linköping, Sweden) by two of the authors (MS, OLJ) by manual calculation with the ellipsoid volume measurement formula ABC/2 in cm^3^ as described before (Barras et al., 2016; Kothari et al., 1996). The ICH score, a simple clinical grading scale that allows risk stratification on presentation with ICH, including the GCS score, age, ICH volume, intraventricular hemorrhage and an infratentorial origin of hemorrhage, was also registered (Hemphill et al., 2001). The mRS was used to determine functional status before and after ICH and was scored by two of the authors (MS, OLJ) (Broderick et al., 2017). Preoperative imaging characteristics were assessed as part of standard clinical practice by a neuroradiologist. These findings were systematically categorized and documented based on their location as either supra- or infratentorial, lobar or non-lobar, whether the hemorrhage affected the basal ganglia or extended into the ventricles (isolated ventricular hemorrhages were excluded). We defined lobar hemorrhages as bleedings occurring in any cerebral hemisphere lobes, affecting cortical or subcortical areas, but explicitly excluding deep hemispheric and infratentorial regions. Non-lobar hemorrhages were defined as bleedings affecting deeper hemispheric structures such as the basal ganglia, thalamus, internal capsule, brainstem, or an infratentorial location. Additionally, the occurrence of hydrocephalus and the spot sign were documented. Vital signs as systolic blood pressure >160 mmHg, tachycardia (>100 bpm), bradycardia (<60 bpm) and hypoxia (sO2 <90 %) were also documented.

Surgical decisions were not part of a study protocol and were made by the on-call senior neurosurgeon. Surgery was typically performed on patients with good pre-admission functional status (mRS 0–3) and space occupying sICH causing reduced consciousness (GCS ≤12). Additionally, next of kin perspectives and potential comorbidities affecting outcomes were taken into consideration. In most cases the hemorrhage is evacuated via craniotomy, the placement of an external ventricular drain (EVD) was indicated when hydrocephalus or relevant amount of intraventricular blood were present. Preoperative anticoagulant reversal was routinely performed in addition to full medical and intensive care.

### 2.2 Outcomes of interest

The primary outcome was functional status three months post-surgery, assessed using the Modified Rankin Scale (mRS) both before ICH and three months after surgery (Broderick et al., 2017). We dichotomized the modified Rankin Scale (mRS) at three months after surgery into two categories: good outcome (mRS 0–3) or poor outcome (mRS 4–6).

The secondary aim of the study was the identification of risk factor for poor outcome (mRS ≥4).

### 2.3 Statistical analysis

IBM SPSS Statistics 28.0 software (IBM, Armonk, New York, USA) was used for statistical analysis. Data was described by standard statistics, using absolute and relative frequencies for categorical variables and median and mean, and range for continuous variables. Cases with missing information about one variable were only excluded from the corresponding statistical analyses but not from the entire study. Univariate logistic regression modeling and chi-square test were used for continuous and categorial variables, respectively. ROC analysis was used to determine cut-off values for continuous variables. All variables that were statistically significant in univariate analysis were entered into a multivariable logistic regression model. Odds ratios (OR) and the corresponding 95 % confidence intervals (CIs) were obtained. We used the β-coefficients of this model to develop a risk score for the prediction of poor and assigned points proportional to the coefficient as described before (Lohmann et al., 2021b; Schipmann et al., 2023). Cases were divided into three categories: low-, intermediate-, and high-risk, corresponding to the total scores. Appropriate cut-off values were determined using the "closest to top left" method. To assess the overall performance of the total score and discrimination between the different risk groups, receiver operating characteristic (ROC) curves were generated. An area under the curve (AUC) above 0.7 reflects reasonable discrimination, while an AUC above 0.8 indicates good discrimination. A probability value less than 0.05 was considered statistically significant throughout the whole analyses. All reported p values are two-sided.

### Ethics approval

This study was conducted on assignment from the department management as a quality control study. Thus, it has been carried out in accordance with Norwegian regulations (Personal Data Act (article 6.1.e and article 9.2.i and the Specialist Healthcare Act §§ 3-4a), without the involvement of the Regional Ethical Committee.

## 3 Results

### 3.1 Study population and demographic data

**Fig. 1:**
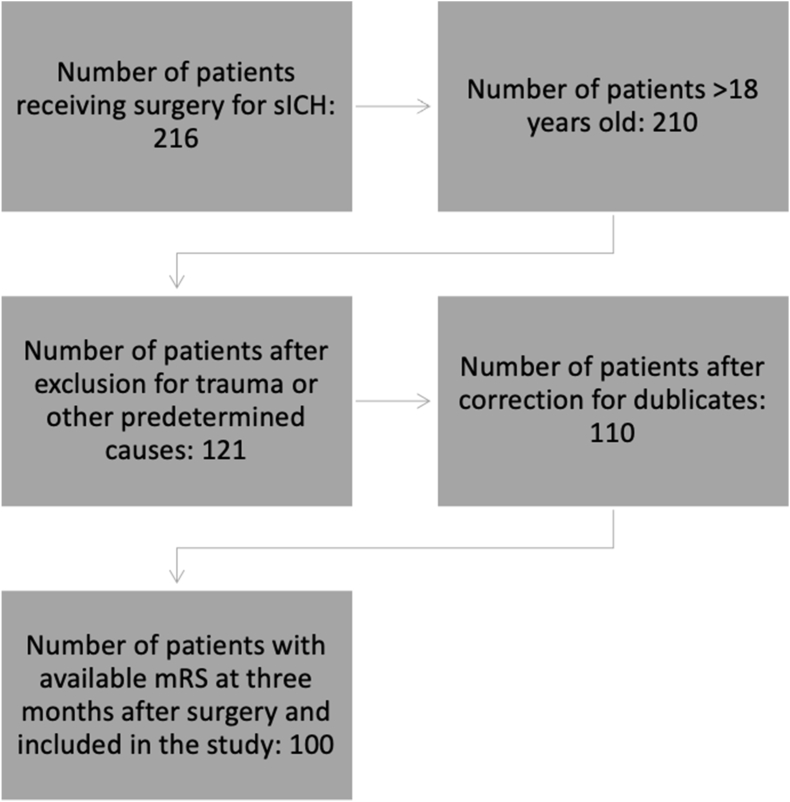
We identified 216 patients who had undergone surgery for spontaneous intracerebral hemorrhage (sICH) during the study period. Only patients aged 18 years and above were included in the analysis. Exclusion criteria comprised patients presenting with ICH resulting from trauma or secondary to cerebral aneurysms, arteriovenous malformations (AVMs), cavernomas, or other identifiable causes. Eleven patients appeared in our records multiple times (due to reoperation or errors in our surgical database) and were therefore excluded after their initial surgical procedure. We included only patients with available information on the Modified Rankin Scale (mRS) at three months post-surgery.

We identified 216 patients who had undergone surgery for sICH during the study period. One hundred patients fulfilled the inclusion criteria and were included in the study (Fig. 1)

The baseline characteristics are presented in Table 1. Most patients were male (56 %), and the mean age was 65.5 years (range: 29–86). Arterial hypertension was the most common comorbidity. Oral anticoagulation was used of 16 % of the patients, whereas 29 % were under treatment with anti-platelet agents. All patients had an mRS score of 3 or lower before manifestation of sICH.

**Table 1:** Baseline characteristics of 100 included cases.

		All patients n (%)	mRS 0-3	mRS 4-6	P-value
			n (%)	n (5)	
			n = 46	n = 54	
Baseline characteristics					

**Age**	Mean, range	65.49 (29–86)	62.88 (29–86)	67.71 (48–84)	0.121
	<60 y	25 (25%)	16 (34.8%)	9 (16.7%)	**0.037**
	≥60 y	75 (75%)	30 (65.2%)	45 (83.3%)	
**Sex**	Male	56 (56%)	26 (56.5%)	30 (55.6%)	0.782
	Female	44 (44%)	20 (43.5%)	24 (44.4%)	0.923
**Secondary diagnoses**	Alcohol abuse	5 (5%)	2 (4.3%)	3 (5.6%)	
	Arterial hypertension	45 (45%)	24 (52.2%)	21 (38.9%)	0.183
	Hypercholesterolemia	9 (9%)	3 (6.5%)	6 (11.1%)	0.424
	Smoking	14 (14%)	8 (17.4%)	6 (11.1%)	0.367
	Hemophilia	2 (2%)	1 (2.2%)	1 (1.9%)	0.909
	Thrombocytopenia	2 (2%)	0 (0%)	2 (3.7%)	0.187
	Cerebral amyloid angiopathy	4 (4%)	3 (6.5%)	1 (1.9%)	0.235
	Diabetes mellitus	15 (15%)	6 (13%)	9 (16.7%)	0.613
	Liver disease	4 (4%)	2 (4.3%)	2 (3.7%)	0.870
	Hematological disorder	5 (5%)	4 (8.7%)	1 (1.9%)	0.118
	Atrial fibrillation	9 (9%)	1 (2.2%)	8 (14.8%)	**0.028**
	Previous stroke	14 (14%)	3 (6.5%)	11 (20.4%)	**0.047**
	Any vascular risk factor	21 (21%)	9 (19.6%)	12 (22.2%)	0.745
	Any bleeding disorder	4 (4%)	1 (2.2%)	3 (5.6%)	0.390
**Use of medicine**	Oral anticoagulation (OAC)	16 (16%)	3 (6.5%)	13 (24.1%)	**0.017**
	Anti-platelet agents	29 (29%)	11 (23.9%)	18 (33.3%)	0.301
	Combination OAG and platelet inhibitors	4 (4%)	0 (0%)	4 (7.4%)	0.050
	Any medication	76 (76%)	37 (80.4%)	39 (72.2%)	0.338
	Recent thrombolysis for suspected stroke	4 (4%)	3 (6.5%)	1 (1.9%)	0.235
**mRS before hemorrhage**	0	26 (26%)	12 (26.1%)	14 (25.9%)	
	1	41 (41%)	22 (47.8%)	19 (35.2%)	0.146
	2	24 (24%)	11 (23.9%)	13 (24.1%)	
	3	9 (9%)	1 (2.2%)	8 (14.8%)	
**Initial diagnosis**					

**Neurological findings**	Seizures	1 (1%)	0 (0%)	1 (1.9%)	0.354
	Motor/sensory deficit	60 (60%)	25 (54.3%)	35 (64.8%)	0.287
	Aphasia	28 (28%)	11 (23.9%)	17 (31.5%)	0.401
**GCS at admission**	3–12	44 (44%)	15 (32.6%)	29 (53.7%)	**0.034**
	13–15	56 (56%)	31 (67.4%)	25 (46.3%)	
**Vital signs**	Syst. BP ≥ 160 mmHg	54 (55.1%)	22 (48.9%)	32 (60.4%)	0.255
	Tachycardia (>100bpm)	7 (7.3%)	3 (6.7%)	4 (7.8%)	0.835
	Bradycardia (<60bpm)	9 (9.4%)	4 (8.9%)	5 (9.8%)	0.878
	Hypoxia (sO2<90%)	3 (3.2%)	1 (2.3%)	2 (3.9%)	0.661
**Imaging**	Hydrocephalus	24 (24%)	7 (15.2%)	17 (31.5%)	0.058
	Midline shift	75 (75%)	36 (78.3%)	39 (72.2%)	0.487
	Mass effect	91 (91%)	42 (91.3%)	49 (90.7%)	0.922
	Herniation	21 (21%)	6 (13%)	15 (27.8%)	0.071
	Spot sign	33 (33%)	13 (28.3%)	20 (37%)	0.352
	Intraventricular blood	52 (52%)	15 (32.6%)	37 (68.5%)	**<0.001**
**Localization**	Supratentorial	85 (85%)	42 (91.3%)	43 (79.6%)	0.103
	Infratentorial	15 (15%)	4 (8.7%)	11 (20.4%)	
	Basal ganglia	26 (26%)	7 (15.2%)	19 (35.2%)	**0.023**
	Lobar	53 (53%)	28 (60.9%)	25 (46.3%)	0.206
	Eloquent	44 (44%)	20 (43.5%)	24 (44.4%)	0.923
	Motor cortex	24 (24%)	6 (13%)	18 (33.3%)	**0.018**
**Hemorrhage volume**	<30 ml	18 (18%)	10 (21.7%)	8 (14.8%)	0.429
	30–60 ml	43 (43%)	21 (45.7%)	22 (40.7%)	
	>60 ml	39 (39%)	15 (32.6%)	24 (44.4%)	
**ICH score**	0	3 (3%)	3 (6.5%)	0 (0%)	**0.004**
	1	17 (17%)	13 (28.3%)	4 (7.4%)	
	2	30 (30%)	16 (34.8%)	14 (25.9%)	
	3	40 (40%)	11 (23.9%)	29 (53.7%)	
	4	9 (9%)	3 (6.5%)	6 (11.1%)	
	5	1 (1%)	0 (0%)	1 (1.9%)	
**Surgery**					

**Time symptom onset - surgery**	<12 h	75 (75%)	31 (67.4%)	44 (81.5%)	0.075
	12h–24h	13 (13%)	10 (21.7%)	3 (5.6%)	
	>24h–72h	7 (7%)	2 (4.3%)	5 (9.3%)	
	>72h	5 (5%)	3 (6.5%)	2 (3.7%)	
**Preoperative anticoagulant reversal**	(only relevant patients)	25 (61%)	10 (21.7%)	15 (27.8%)	0.323

**Surgical approach**	Craniotomy	98 (98%)	45 (97.8%)	53 (98.1%)	0.909
	Craniectomy	2 (2%)	1 (2.2%)	1 (1.9%)	0.909
	Additional EVD	7 (7%)	1 (2.2%)	6 (11.1%)	0.081
**Cutting suture time**	Mean (range)	94.63 (23–302)	89.3 (23–302)	99.17 (48–225)	0.062

**Surgical outcome**					

**mRS 3 month after surgery**	0	1 (1%)	1 (2.2%)		**<0.001**
	1	10 (10%)	10 (21.7%)		
	2	12 (12%)	12 (26.1%)		
	3	23 (23%)	23 (50%)		
	4	26 (26%)		26 (48.1%)	
	5	5 (5%)		5 (9.3%)	
	6	23 (23%)		23 (42.6%)	

The majority of patients presented with motor or sensory deficits at admission (60 %) and with a GCS between 13 and 15 (56 %). Hemorrhage location was predominantly supratentorial (85 %). Hydrocephalus was radiologically identified in one-fourth of the cases (24 %), and intraventricular extension of hemorrhage was present in half (52 %) of the patients. A positive spot sign was identified in 33 % of the patients. Eighty-eight patients (88 %) underwent surgical treatment within 24 h after symptom onset, with 75 % within the first 12 h.

Table 1. Baseline characteristics of the 100 included cases and stratified into patients with mRS 0–3 and 4–6 three months after surgery.

### 3.2 Outcome

**Fig. 2:**
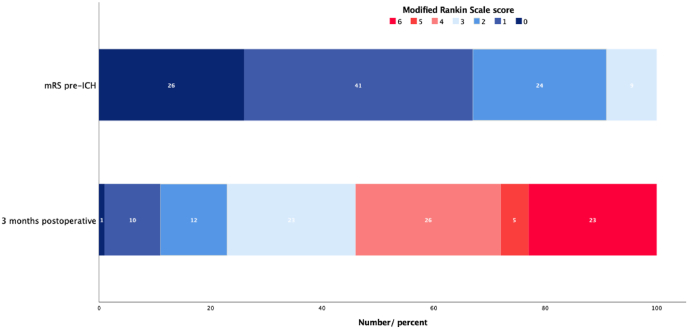
A significant difference between mRS before and after surgery for sICH (p = 0.037).

More than half of the patients (54 %) had an mRS score of 4 or higher three months postoperative, with 23 patients (23 %) having a score of 6 (death) (Fig. 2). There was a significant difference between mRS before and after surgery for ICH (p = 0.037).

### 3.3 Risk factors for unfavorable outcome

We evaluated the association of various variables with unfavorable outcome (mRS ≥4 after three months). Univariate analysis revealed age ≥60 y, atrial fibrillation, previous stroke, oral anticoagulation, GCS at admission between 3 and 12, presence of intraventricular blood, hemorrhage located in the basal ganglia or motor cortex, ICH score as risk factors for unfavorable outcome (Table 1).

### 3.4 Risk factors predicting unfavorable outcome

Independent risk factors for poor outcome (mRS ≥4) were age ≥60 y (OR 7.8, 95 % CI 1.684–36.3, *p* = 0.009), oral anticoagulation (OR 10.4, 95 % CI 1.495–72.665, *p* = 0.018), hemorrhage in the basal ganglia (OR 18.5, 95 % CI 3.398–100.717, *p* = <0.001) or motor cortex (OR 8.6, 95 % CI 2.134–34.973, *p* = 0.003) (Table 2).

**Table 2:** Independent risk factors for poor outcome (mRS ≥4).

		OR	95 %-CI		p-value
**Age**	<60	Ref			
	≥60	7.8	1.684	36.300	0.009
**Oral anticoagulation**	no	Ref	1.495	72.665	0.018
	yes	10.4			
**Hemorrhage in basal ganglia**	no	Ref	3.398	100.717	<0.001
	yes	18.5			
**Hemorrhage motor cortex**	no	Ref			
	yes	8.6	2.134	34.973	0.003

OR= Odds Ratio, 95 %-CI: 95 % confidence interval.

Table 2. Multivariate logistic regression model predicting the risk for mRS at three months postoperatively. The OR (Odds Ratio) was calculated. Only statistically significant results (p < 0.05) are presented.

**Fig. 3:**
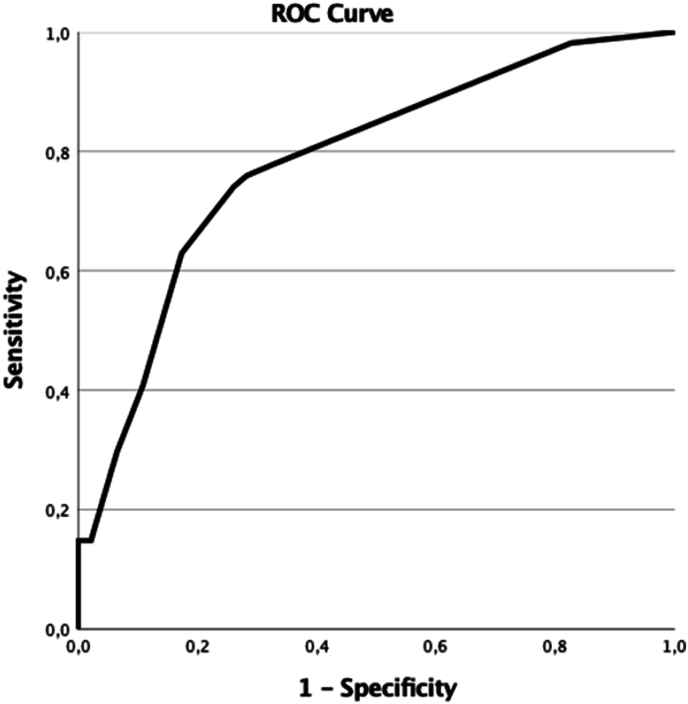
ROC curve for the total score: AUC 0.783 (95 % CI 0.693–0.874, p < 0.001).

A HeMAtOma score was calculated incorporating the four independent risk factors to predict the risk of unfavorable outcome (Table 3). ROC analysis for the total score demonstrated reasonable discrimination with an AUC of 0.783 (95 % CI 0.693–0.874, p < 0.001, Fig. 3). To facilitate clinical application of the score, patients were categorized into risk groups. A cut-off of 21 was established to distinguish between low- and intermediate-risk patients, yielding a sensitivity of 0.778 and a specificity of 0.674.

**Table 3:** HeMAtOma score.

	Category	β-coefficient	p-value	Score
**Hemorrhage in basal ganglia**	no	Ref	<0.001	+29
	yes	2.918		
**Hemorrhage Motor cortex**	no	Ref		
	yes	2.156	0.003	+22
**Age**	<60	Ref	0.009	+21
	≥60	2.056		

**Oral anticoagulation**	no	Ref	0.018	+23
	yes	2.344		
**Total**				
Out of max score of 95

A score exceeding 50 was associated with a 43-fold increased likelihood of an mRS score ≥4 after three months (Table 4). ROC analysis revealed fair discrimination among the three risk groups with an AUC of 0.688 (95 % CI 0.563–0.773, p = 0.002).

**Table 4:** Classification of the risk groups based on the score and calculation of the odds ratios for the risk of mRS ≥4 three months after surgery.

Risk group	Score	Number of cases (n)	mRS ≥4 (n,%)	OR	95 %-CI	p-value
**Low-risk**	0	9	1 (11.1 %)	Ref		
**Intermediate-risk**	21–44	72	37 (51.4 %)	8.46	1.005–71.138	0.049
**High-risk**	50–95	19	16 (84.2 %)	42.67	3.805–478.419	0.002

Table 3: Calculation of the score to predict the risk for poor outcome with mRS ≥4 three months after surgery.

Classifying patients into different risk groups revealed an incidence of mRS score of ≥4 of 11.1 % in the low-risk, 51.4 % in the intermediate-risk and 84.2 % in the high-risk group, respectively (p < 0.001) (Table 4).

Table 4: Classification of the risk groups based on the score and calculation of the odds ratios for the risk of mRS ≥4 three months after surgery. ROC analysis revealed an AUC of 0.668 (p = 0.004). For example, a 70 year old patient with hemorrhage in the basal ganglia and use of oral anticoagulants falls into the high-risk group with a score of 43 (see Table 3) and has and Odds ratio of 42.67 for a mRS ≥4.

## 4 Discussion

We present a retrospective analysis of patients who underwent surgical treatment for sICH, evaluating their functional outcomes three months post-surgery and developed a clinical score to help identify those who could benefit from surgical intervention.

The primary outcome of our study was the functional outcome three months following surgery. We dichotomized functional outcome in favorable (mRS 0–3) and unfavorable (mRS 4–6). This classification differentiates between patients who retain some degree of functional independence, including the ability to ambulate and perform basic self-care tasks with potential assistance for daily activities, and those who are fully dependent on caregivers for all aspects of daily living or have died.

At the three-month follow-up, despite careful selection of the best candidates, the majority (54 %) of our patients demonstrated unfavorable outcomes (mRS 4–6), with 23 % (n = 23) achieving a modified Rankin Scale (mRS) score of 6, corresponding to a 90-days mortality-rate of 23 %. Our findings are in line with previous study (Abulhasan et al., 2023), illustrating high rates of disability and mortality following sICH, despite comprehensive medical and surgical intervention. These outcomes also reflect the results from major randomized controlled trails that fail to support early surgery for sICH compared to medical treatment alone (Hanley et al., 2019; Mendelow et al., 2005, 2013).

However, as recent guidelines suggest, surgical treatment may be advantageous for specific patient groups (Greenberg et al., 2022b). The ENRICH trial demonstrated improved functional outcomes at 180 days post-intervention for patients who underwent minimally invasive hemorrhage evacuation, particularly in cases of lobar hemorrhages. Additional studies have indicated that surgical management of sICH is associated with reduced 12-month mortality rates, although it does not significantly increase the likelihood of survival without permanent disability (Luostarinen et al., 2020). Abulhasan et al. showed that lifesaving surgery for sICH did not significantly alter mortality or influence functional outcome in patients overall. However, a subgroup analysis revealed that patients with ICH scores of 3 and 4 experienced reduced 30-day mortality rate when treated with a comprehensive medical and surgical approach. Nonetheless, this survival benefit was not sustained at 90 days, and long-term outcomes remained poor (Abulhasan et al., 2023).

These findings highlight the complexities involved in managing sICH and underscore the necessity for meticulous patient selection when considering surgical intervention. Understanding the risk factors for poor outcomes is vital. In our cohort, independent risk factors for unfavorable outcomes included being aged 60 or older, using oral anticoagulants, and having hemorrhages located in the basal ganglia or motor cortex.

Higher age has been shown as an independent risk factor for poor outcome in many studies (Hemphill et al., 2001; Radholm et al., 2015; Zhang et al., 2022). The involvement of the basal ganglia and motor cortex, as highly functional areas, resulting in severe neurological deficits regarding motor function were risk factors for unfavorable outcome both in our study and others (Mendelow et al., 2013; Zhang et al., 2022). The use of oral anticoagulants carries a higher risk both for spontaneous hemorrhages in various locations (Kocjan et al., 2024) and has been identified as an independent risk factor for unfavorable outcome in our study, even with timely reversal of anticoagulation (Oie et al., 2018).

Other known risk factors noted in the literature only reached significance in univariate analyses in our study. These included low GCS scores (Hemphill et al., 2001; Oie et al., 2018; Troberg et al., 2018; Zubkov et al., 2008) and the presence of intraventricular hemorrhage (Hemphill et al., 2001; Li et al., 2020; Mendelow et al., 2013; Oie et al., 2018).

Admitting a patient with sICH constitutes an emergency that requires prompt decision-making about whether to fully utilize medical and surgical resources or to shift to palliative care alone. This can be particularly challenging when there is limited clinical information available about the patient, especially in cases of rapid deterioration, highlighting the need for straightforward tools to aid decision-making.

Risk scoring systems can assist clinicians across various medical fields in estimating the probability of specific outcomes based on individual patient variables. These scores help categorize patients into different risk groups, facilitating clinical decision-making and optimizing resource allocation (Han et al., 2018; Lohmann et al., 2021a; Tisdale et al., 2013).

A commonly used grading system to predict outcome in patients with sICH is the well-established ICH score (Hemphill et al., 2001). The score consists of five components: Glasgow Coma Scale (GCS) score, age, ICH volume, presence of intraventricular hemorrhage, and infratentorial origin of the hemorrhage. It has been validated for both, predicting the 30-day mortality and long-term functional outcomes up to 12 months post-ICH (Hemphill et al., 2001, 2009). Univariate analysis revealed the ICH score as a risk factor for unfavorable outcome in our study. We calculated the ICH score for all the patients prior to surgery, discovering that 90 % had an ICH score of 3 or lower. This finding demonstrates our approach of early identification of high-risk patients and careful selection of surgical candidates.

Based on the independent risk factors we identified, we developed the HeMAtOma score to predict functional outcomes three months after surgery. The risk factors included in this score are easily obtainable even in emergency situations: radiological findings (specifically the location of the hemorrhage in the basal ganglia or motor cortex), patient age, and the use of oral anticoagulants.

Patients can be stratified into different risk groups (low-risk, intermediate-risk and high-risk) based on their scores. Those in the high-risk group have a 43-fold increased likelihood of experiencing an unfavorable outcome, defined as a modified Rankin Scale (mRS) score of 4 or higher, three months post-treatment. For example, nearly 85 % of high-risk patients have an mRS score of 4 or greater three months after surgery. Conversely, if a patient does not meet any criteria for the score, there is a 90 % chance of a favorable outcome following surgery, making surgical intervention the recommended course of action.

Our score aims to enhance clinical decision-making while improving communication with patients and their families regarding further treatment options.

### 4.1 Limitations

Several limitations of this study should be acknowledged. As with any retrospective study, it bears inherent limitations. Notably, in cases lacking documentation of the mRS score, two authors relied on information from digital patient records to assign scores. Additionally, due to insufficient follow-up data, we had to exclude half of the patients, which may have biased our results. Most of these patients reside in a different healthcare district that limits our access to follow-up data. We also do not have follow-up data extending beyond three months post-surgery, precluding an evaluation of long-term outcomes. A further relevant limitation is selection bias, as the decision to perform surgery (and indirectly, inclusion in the study) was based on the individual judgment of the attending surgeon. We have clearly specified that we only operated on patients with good functional status prior to sICH, which limits our conclusions to this specific subgroup of previously well-functioning patients.

Finally, the developed score requires both internal and external validation.

## 5 Conclusions

Outcome regarding functional status and mortality remains poor despite comprehensive medical and surgical treatment for sICH. We identified age, the presence of a hemorrhage in the basal ganglia or motor cortex, and the use of oral anticoagulants as independent risk factors for unfavorable outcomes after surgery. From this, we created the HeMAtOma score to predict functional outcomes at three months post-surgery. This score may assist clinicians in emergency situations by accurately identifying patients who would benefit from surgical intervention. Further prospective studies are necessary to validate the score both internally and externally.

## Author contributions

Conception and design: M.S, O.L.J, Ø.V.T, R.M,; acquisition of data and analysis: M.S, O.L.J, S.S.M; writing – original draft preparation: M.S, O.L.J, Ø.V.T, S.S.M, R.M; writing – reviewing and/or editing of manuscript: M.S, O.L.J, Ø.V.T, S.S.M, R.M. All authors have read and agreed to the published version of the manuscript.

## Ethical approval

The study protocol was exempt from review by the Regional Ethical Committee of Western Norway as it classifies as a quality improvement study, which only requires permission from the local hospital. This arrangement is regulated by Norwegian law under The Personal Data Act (personopplysningsloven) article 6.1.e, and article 9.2.i, and the Specialist Healthcare Act (spesialisthelsetjenesteloven) §§ 3-4a.

## Disclosure

Conflict of Interest Statement.

The authors whose names are listed below certify that they have no affiliations with or involvement in any organization or entity with any financial interest (such as honoraria; educational grants; participation in speakers’ bureaus; membership, employment, consultancies, stock ownership, or other equity interest; and expert testimony or patent-licensing arrangements), or non-financial interest (such as personal or professional relationships, affiliations, knowledge or beliefs) in the subject matter or materials discussed in this manuscript.

## Funding

No funding was received for this research.

## Declaration of competing interest

The authors declare no conflict of interest.
